# The Role of miR-330-3p/PKC-α Signaling Pathway in Low-Dose Endothelial-Monocyte Activating Polypeptide-II Increasing the Permeability of Blood-Tumor Barrier

**DOI:** 10.3389/fncel.2017.00358

**Published:** 2017-12-19

**Authors:** Jiahui Liu, Libo Liu, Shuo Chao, Yunhui Liu, Xiaobai Liu, Jian Zheng, Jiajia Chen, Wei Gong, Hao Teng, Zhen Li, Ping Wang, Yixue Xue

**Affiliations:** ^1^Department of Neurobiology, College of Basic Medicine, China Medical University, Shenyang, China; ^2^Key Laboratory of Cell Biology, Ministry of Public Health of China, and Key Laboratory of Medical Cell Biology, Ministry of Education of China, China Medical University, Shenyang, China; ^3^Department of Neurosurgery, Shengjing Hospital of China Medical University, Shenyang, China; ^4^Liaoning Clinical Medical Research Center in Nervous System Disease, Shenyang, China; ^5^Key Laboratory of Neuro-oncology in Liaoning Province, Shenyang, China

**Keywords:** EMAP-II, BTB, miR-330-3p, PKC-α, glioma

## Abstract

This study was performed to determine whether EMAP II increases the permeability of the blood-tumor barrier (BTB) by affecting the expression of miR-330-3p as well as its possible mechanisms. We determined the over-expression of miR-330-3p in glioma microvascular endothelial cells (GECs) by Real-time PCR. Endothelial monocyte-activating polypeptide-II (EMAP-II) significantly decreased the expression of miR-330-3p in GECs. Pre-miR-330-3p markedly decreased the permeability of BTB and increased the expression of tight junction (TJ) related proteins ZO-1, occludin and claudin-5, however, anti-miR-330-3p had the opposite effects. Anti-miR-330-3p could enhance the effect of EMAP-II on increasing the permeability of BTB, however, pre-miR-330-3p partly reversed the effect of EMAP-II on that. Similarly, anti-miR-330-3p improved the effects of EMAP-II on increasing the expression levels of PKC-α and p-PKC-α in GECs and pre-miR-330-3p partly reversed the effects. MiR-330-3p could target bind to the 3′UTR of PKC-α. The results of *in vivo* experiments were similar to those of *in vitro* experiments. These suggested that EMAP-II could increase the permeability of BTB through inhibiting miR-330-3p which target negative regulation of PKC-α. Pre-miR-330-3p and PKC-α inhibitor decreased the BTB permeability and up-regulated the expression levels of ZO-1, occludin and claudin-5 while anti-miR-330-3p and PKC-α activator brought the reverse effects. Compared with EMAP-II, anti-miR-330-3p and PKC-α activator alone, the combination of the three combinations significantly increased the BTB permeability. EMAP-II combined with anti-miR-330-3p and PKCα activator could enhance the DOX’s effects on inhibiting the cell viabilities and increasing the apoptosis of U87 glioma cells. Our studies suggest that low-dose EMAP-II up-regulates the expression of PKC-α and increases the activity of PKC-α by inhibiting the expression of miR-330-3p, reduces the expression of ZO-1, occludin and claudin-5, and thereby increasing the permeability of BTB. The results can provide a new strategy for the comprehensive treatment of glioma.

## Introduction

In the glioma, blood-tumor barrier (BTB) is the key factor to restrict the delivery of large therapeutic molecules into the tumor tissues, which affects the efficacy of chemotherapy. BTB has different characteristics from the BBB, which results from damage caused by glioma cells development. Although the permeability of BTB is slightly higher than BBB, BTB still poses a major hurdle to anticancer drug delivery to tumors (Black and Ningaraj, 2004). Therefore, selectively enhancing the permeability of BTB is an urgent problem to be solved to deliver anti-tumor drugs into tumor tissues effectively and to improve the therapeutic effect of glioma.

Endothelial monocyte-activating polypeptide-II (EMAP-II), which is synthesized by its precursor proEMAP-II, can regulate the function of endothelial cells and monocytes (Shalak et al., 2007). At present, it has been found that EMAP-II has many functions on regulating cell function. In the development of hyperoxia-induced lung disease of prematurity, EMAP-II can mediate macrophage migration (Lee et al., 2016). EMAP-II is increased in children and adolescents with type I diabetes, which are related to micro-vascular complications (Adly et al., 2015). EMAP-II can induce C6 glioma cells apoptosis via the mitochondrial pathway (Liu L. B. et al., 2015). EMAP-II alone or combined with rapamycin can inhibit the viability, migration and invasion of glioma cells through inducing autophagy (Ma et al., 2015; Chen et al., 2016). Studies have reported that low-dose EMAP-II (0.05 nM) selectively increases the permeability of BTB via the cAMP/PKA signaling pathway and the PKC-ζ/PP2A signaling pathway (Li et al., 2015a,c). *In vitro* BTB model, low-dose EMAP-II can bind to α-ATP synthase on BMECs surface and open tight junction (TJ) to selectively increase the permeability of BTB by significantly decreasing the protein expression levels of TJ-related proteins ZO-1, occludin and claudin-5 (Xie et al., 2010; Li et al., 2011). Other studies have demonstrated that protein kinase C (PKC) has distinct effects on the dynamic changes in TJ and the permeability of endothelial cells. PKC activation resulting in phosphorylation and redistribution of TJ related proteins, contributes to regulating the TJ (Sjö et al., 2010). It was found that EMAP-II could increase the permeability of BTB model *in vitro* by activating PKC, and the effects of EMAP-II on BTB permeability were significantly diminished by H7, the inhibitor of PKC (Li et al., 2012b). The above studies suggest that PKC plays an important regulatory role in EMAP-II increasing the permeability of BTB. However, the mechanism of EMAP-II regulating the expression and activity of PKC is unclear.

MicroRNAs are a class of small non-coding RNAs with 18–25 nucleotides in length. MiRNAs exert effects on regulating target genes by post-transcription, and regulate the biological functions of many kinds of cells in physiological and pathological processes (Zimmerman and Wu, 2011). MircoRNA-330 (miR-330) gene was discovered by Weber (2005), located in 19Q12.32, and expressed in a variety of tissues with different functions (Lee et al., 2009; Goyal et al., 2010; Hodzic et al., 2011). The sequence for miR-330 lies in the first intron of *Eml2*, a microtubule-associated protein, which alters the assembly dynamics of microtubules. It is possible that miR-330 is transcribed from its host gene, *Eml2*. Conversely, it may inhibit the expression of *Eml2* through binding the site of *Eml2* 3′-UTR. Study also found that miR-330 transcription could be independent of *Eml2* as potential RNA *Pol*III-regulatory elements associated with miR-330 have been found (Medrano et al., 2012). The major subtypes of miR-330 include miR-330-5p and miR-330-3p. MiR-330-3p was over-expressed in non-small cell lung cancer (NSCL) cell lines A549 and H23, which controls cell proliferation by targeting early growth response 2 (Liu X. et al., 2015). MiR-330-3p was enriched in breast cancer and it targeted CCBE1 to promote the invasion and metastasis of breast cancer cell lines (Mesci et al., 2017). Meng et al. (2015) found that miR-330-3p was highly expressed in esophageal squamous cell carcinoma (ESCC) tumor tissues and ESCC cell lines, overexpression of miR-330-3p remarkably enhanced ESCC cell proliferation, survival, migration and invasion *in vitro*. Meanwhile, we have demonstrated miR-330 could promote cellular proliferation, migration and invasion of glioma U87 and U251 cells and glioma stem cells by targeting SH3GL2 (Qu et al., 2012; Yao et al., 2014). The above studies indicate that miR-330-3p acts as oncogenes. While, other studies found miR-330-3p was lowly expressed in gastric cancer cell lines and tissues, and over-expression of miR-330-3p could inhibit gastric cancer progression through targeting MSI1 (Guan et al., 2016). MiR-330 was able to induce apoptosis of prostate cancer cells through inhibiting the effect of E2F1 on Akt phosphorylation (Lee et al., 2009). These suggest that miR-330-3p acts as a tumor suppressor gene in gastric cancer and prostate cancer. Although miR-330-3p acts as oncogenes in glioma, the effect of miR-330-3p on brain microvascular endothelial cells of glioma has not yet been reported.

This study aims to investigate the endogenous expression of miR-330-3p in glioma microvascular endothelial cells (GECs) and whether the expression of miR-330-3p is regulated by EMAP-II. The aim of further study is on whether EMAP-II affects the permeability of BTB by regulating the expression of miR-330-3p and the effect and possible mechanism of miR-330-3p regulating BTB permeability. In this study, we aim to explore the molecular mechanisms on, associated with low-dose EMAP-II, selectively increasing the permeability of BTB and provide new ideas for comprehensive treatment of glioma.

## Materials and Methods

### Cell Lines and Cell Cultures

The immortalized human brain endothelial cell line hCMEC/D3 (ECs) was provided by Dr. Couraud (Institut Cochin, Paris, France). Cells were cultured in endothelial basal medium (EBM-2; Lonza, Walkersville, MD, USA), containing 5% fetal bovine serum (FBS, PAA Laboratories GmbH, Pasching, Austria), 1.4 μmol/L hydrocortisone (Sigma-Aldrich, St. Louis, MO, USA), 1% Penicillin-Streptomycin (Life Technologies Corporation, Paisley, UK), 5 μg/mL ascorbic acid (Sigma-Aldrich, St. Louis, MO, USA), 1% chemically defined lipid concentrate (Life Technologies Corporation, Paisley, UK), 1 ng/mL human basic fibroblast growth factor (bFGF, Sigma-Aldrich, St. Louis, MO, USA) and 10 mmol/L HEPES (PAA Laboratories GmbH).

Human glioma cell lines U87 and human embryonic kidney (HEK) 293T cells were obtained from Shanghai Institutes for Biological Sciences Cell Resource Center. Cells were cultured in high glucose Dulbecco’s Modified Eagle Medium (DMEM), which was supplemented with 10% FBS (Gibco, Carlsbad, CA, USA). Cells were cultured in a humidified incubator at 5% CO_2_ and 37°C, and medium was refreshed every 2 days.

### Establishment of an *in Vitro* Blood-Tumor Barrier (BTB) Model

The *in vitro* BTB model was established by co-culturing ECs with U87 cells in Transwell system permeable support systems (0.4 μm pore size; Corning, NY, USA). First, 2 × 10^6^ U87 cells were seeded onto the lower chamber of Transwell inserts. After the U87 cells were confluent, 2 × 10^5^ ECs were placed on the upper chamber of the Transwell inserts. After ECs and U87 cells were co-cultured 96 h, *in vitro* BTB model was established successfully and the GECs which were ECs co-cultured with glioma cells were successfully obtained and used for study.

### Experimental Groups

To test the effect of EMAP-II on the expression of miR-330-3p, the experiments were divided into 5 groups (*n* = 5): (1) EMAP-II 0 h group (cells were treated with EMAP-II for 0 h); (2) EMAP-II 0.5 h group (cells were treated with EMAP-II for 0.5 h); (3) EMAP-II 1 h group (cells were treated with EMAP-II for 1 h); (4) EMAP-II 2 h group (cells were treated with EMAP-II for 2 h); and (5) EMAP-II 4 h group (cells were treated with EMAP-II for 4 h). EMAP-II (Sigma–Aldrich, St. Louis, MO, USA) was dissolved in 0.9% sodium chloride. According to the previous researches, 0.05 nM was selected as the optimal concentration for this study (Li et al., 2012b, 2016; Xie et al., 2012).

The miR-330-3p angomir, miR-330-3p antigomir and their respective NC (GenePharma, Shanghai, China) were transiently transfected into ECs according to the protocols of Lipofectamine 3000 Reagents. To study the effects of miR-330-3p on the permeability of BTB and the expression of TJ related proteins, the experiments were divided into five groups (*n* = 5): (1) Control group (untransfected ECs); (2) pre-NC group (transfected with blank control of miR-330-3p over-expression plasmid); (3) pre-miR-330-3p group (transfected with miR-330-3p over-expression plasmid); (4) anti-NC group (transfected with blank control of miR-330-3p silencing plasmid); and (5) anti-miR-330-3p group (transfected with miR-330-3p silencing plasmid).

To detect the effects of miR-330-3p on EMAP-II affecting the BTB of GECs and regulating PKCα, the experiments were divided into six groups (*n* = 5): (1) Control group; (2) EMAP-II group; (3) pre-miR-330-3p group; (4) pre-miR-330-3p+EMAP-II group; (5) anti-miR-330-3p group; and (6) anti-miR-330-3p+EMAP-II group.

In order to test the effect of PKCα on miR-330-3p affecting the BTB of GECs, the experiments were divided into seven groups (*n* = 5): (1) Control group; (2) pre-miR-330-group; (3) PKCα activator group; (4) pre-miR-330-3p+PKCα activator group; (5) anti-miR-330-3p group; (6) PKCα inhibitor group; and (7) anti-miR-330-3p +PKCα inhibitor group. PKCα activator phorbol-12-myristate-13-acetate (PMA, 0.1 μM) and PKCα inhibitor staurosporine (2 nM) were purchased from Sigma-Aldrich, St. Louis, MO, USA and used in the BTB of GECs.

To further verify the regulation effects of EMAP-II, anti-miR-330-3p and PKCα activators alone or that of the combination on the BTB of GECs, the experiments were divided into five groups (*n* = 5): (1) Control group; (2) EMAP-II group; (3) anti-miR-330-3p group; (4) PKCα activator group; and (5) EMAP-II+anti-miR-330-3p+PKCα activator group.

To study the effects of EMAP-II, anti-miR-330-3p, PKCα activator on DOX inhibiting the cell viabilities and apoptosis of U87 cells, the experiments were divided into four groups (*n* = 5): (1) Control group; (2) EMAP-II+anti-miR-330-3p +PKCα activator; (3) DOX group; and (4) EMAP-II+anti-miR-330-3p+PKCα activator+DOX group.

### Transendothelial Electric Resistance (TEER) Assays

To measure the integrity of the BTB, after *in vitro* BTB models were established, Transendothelial Electric Resistance (TEER) assay was determined with Millicell-ERS apparatus (Millipore, Billerica, MA, USA). TEER values were measured at room temperature after medium exchange to ensure temperature equilibration and the same medium composition during the measurement. Background electrical resistance was subtracted before final resistances were calculated. Electrical resistance was calculated as Ω cm^2^ by multiplying using the surface area of the transwell insert.

### Horseradish Peroxidase (HRP) Flux Measurement

Horseradish Peroxidase (HRP) flux was detected to further study the permeability of the *in vitro* BTB models. After BTB models were established, 1 ml HRP (0.5 μM, Sigma-Aldrich, USA) in serum-free EBM-2 culture medium was added into the upper chamber of the transwell system and 2 ml of culture medium was added into the well. One hour later, the medium in the lower chamber was gathered and the HRP content of the samples were assayed with Microplate Reader (Varioskan Flash, Thermo Scientific). The HRP flux was expressed as pmol passed per cm^2^ surface area per hour.

### RNA Extraction and Quantitative RT-PCR (qRT-PCR)

The expression levels of miR-330-3p and PKCα were measured by Real-Time PCR analysis, which was by means of a 7500 Fast Real-Time PCR System. Total RNA was extracted from cells with TRIzol reagent (Life Technologies Corporation, Carlsbad, CA, USA). Reverse transcription and Real-Time PCR amplification were carried out using Taqman MicroRNA Reverse Transcription Kit and Taqman Universal Master Mix II with the TaqMan MicroRNA Assay of miR-330-3p and U6 (Applied Biosystems, Foster, CA, USA) were used to quantify the miR-330-3p expression. U6 were used as endogenous controls. Fold changes were calculated using relative quantification (2−∆∆Ct) method.

### Western Blot Assays

Ice-cold RIPA buffer (50 mM Tris-HCl, pH 8.0, 150 mM NaCl, 0.1% SDS, 1% NP-40, 0.5% sodium deoxycholate and 1 mM EDTA) were used to lyse the cells and protease inhibitors (10 mg/mL aprotinin, 10 mg/mL phenyl-methylsulfonyl chloride, and 50 mM sodium orthovanadate) or phosphatase inhibitors were added. After incubating on ice for 20 min, the lysates were centrifuged at 17,000 *g* for 30 min at 4°C. The supernatant extracts were quantified by using the BCA protein assay kit (Beyotime Institute of Biotechnology). Equal amounts of protein were further fractionated by SDS-PAGE and electrophoretically transferred to polyvinylidene difluoride membranes. Non-specific bindings were blocked by incubating membranes in Tris-buffered saline-Tween (TBST) containing 5% non fat milk for 2 h, and subsequently incubated with primary antibodies for PKCα and p-PKCα (1:1000, Abcam, Cambridge, MA, USA), ZO-1 (1:500, Life Technologies Corporation, Frederick, MD, USA), occludin and claudin-5 (1:250, Life Technologies Corporation, Frederick, MD, USA). After this, the membranes were incubated with respective HRP conjugated secondary antibodies. Immunoblots were visualized by ECL chemiluminescent detection system. All the protein bands were scanned using Chem Imager 5500 V2.03 software (AlPha Innotech, San Leandro, CA, USA) and the integrated density value (IDV) was calculated by FluorChem 2.0 software.

### Immunofluorescence Assays

GECs on insert filters were fixed with 4% paraformaldehyde for 30 min and blocked by incubation in PBS solution containing 5% BSA for 2 h with room temperature. Cells were incubated with anti-ZO-1, anti-occludin, anti-claudin-5 antibodies (all diluted at 1:50), respectively, at 4°C overnight. Then cells were incubated with fluorophore-conjugated secondary antibodies for 2 h. After the final wash to visualize cell nuclei for 7 min, DAPI was applied to the samples. The staining was analyzed by Olympus DP71 immunofluorescence microscopy (Olympus, Tokyo, Japan) and merged with Chemi Imager 5500 V2.03 software.

### Reporter Vector Construction and Luciferase Assays

TargetScan Human Release 6.2 was used to predict the putative binding site between the 3′UTR of PKCα mRNA and the seed region of miR-330-3p. HEK293 cells were seeded into a 96-well plate and cultured overnight at 37°C. Then cells were co-transfected with the wild-type (Wt) or mutated (Mut) PKCα-3′UTR reporter plasmid (GenePharma, Shanghai, China), and transfected with miR-330-3p angomir or miR-330-3p angomir NC. Luciferase assays were performed 48 h later using the Dual-Luciferase Reporter Assay System (Promega, Beijing, China).

### Cell Proliferation Assay

GECs were seeded in 96-well plates at a density of 2000 cells/well, and cell viability was detected using the Cell Counting Kit-8 (CCK-8) assay (Beyotime Institute of Biotechnology) according to the instructions provided by the manufacturer. Ten microliter of CCK8 was added into each well after 48 h. Then incubated at 37°C for 2 h and absorbance was measured at 450 nm.

### Apoptosis Was Detected by Flow Cytometry

Annexin V-PE/7-AAD staining apoptosis detection kit (Southern Biotech, Birmingham, AL, USA) was carried out to evaluate apoptosis according to the manufacturer’s instructions. The cells were harvested and stained with Annexin V-PE and 7-AAD after the cells washed two times with 4°C PBS. Then acquired the cells by flow cytometry (FACScan, BD Biosciences, San Jose, CA, USA) and analyzed by CELL Quest 3.0 software.

### Lentivirus Vector Construction and Infection

According to miRBase database, the double-stranded oligonucleotide sequence containing human pre-miR-330-3p was chemically synthesized and ligated into pcDNA6.2-GW/EmGFP-miR expression vector by T4 DNA ligase. After sequencing, the recombinant plasmid pcDNA6.2-GW/ EmGFP-miR-330-3p was recombined into the lentiviral vector by Gateway Vector Kit (Life Technologies Corporation, Carlsbad, CA, USA), named pLenti6.3-miR-330-3p. Lentivirus was generated in 293FT cells using the ViraPower Packaging Mix. After infection, the miR-330-3p-overexpression (miR-330-3p (+)) stable expressing cells were picked.

### Orthotopic Xenograft Model

The male BALB/c athymic nude mice (*n* = 5, 4–6 weeks old) purchased from the Vital River Company (Beijing, China). All animal experiments were carried out under the approval of the Administrative Panel on Laboratory Animal Care of China Medical University. The animals were free to autoclaved food and water during the study.

The infected U87 glioma cells were harvested at log phase by centrifugation. The mice were anesthetized with 10% chloral hydrate (3.5 ml/kg, i.p.), U87 glioma cells suspension was injected through a Hamilton syringe and the stereotaxic apparatus. The coordinates were 3 mm lateral, 1 mm anterior to the bregma and 4 mm deep from the skull surface in the right brain. After 8 days later, mice were injected of EMAP-II (80 ng/kg) or 0.9% sodium chloride into the tumor-bearing mice brains for 1 h via the same needle track where glioma cells were implanted into the mice.

The mice were divided into four groups (*n* = 5): (1) control group, U87 cells (without miR-330-3p stable expressing) treated with 0.9% sodium chloride; (2) EMAP-II group, U87 cells (without miR-330-3p stable expressing) treated with EMAP-II; (3) miR-330-3p (+) group, miR-330-3p stable expressing U87 cells; and (4) EMAP-II + miR-330-3p (+) group, miR-330-3p stable expressing U87cells treated with EMAP-II.

### Evans Blue (EB) and qRT-PCR Assays

The BTB permeability was quantitatively evaluated by extravasation of evans blue (EB) as a marker (Liu et al., 2008). Briefly, 2% EB in saline (2 mg/kg) was injected intravenously for 2 h before ventricular perfusion. After EMAP-II was injected into the tumor-bearing mice brains via the same needle track for 1 h, all the mice were deeply anesthetized with 10% chloral hydrate and perfused with heparinized saline through the cardiac ventricle until colorless perfusion fluid was obtained from the atrium. After mice were euthanized and the brains were removed, the hemispheres of brain were separated along the interhemispheric plane. Then both hemispheres were weighed and put into formamide (1 ml/100 mg) for 24 h at 60°C. The supernatant was obtained, and its optical density was determined by spectrophotometer at 620 nm (Shimadzu, Japan). The quantitative calculation of the dye content in the brain was based on the standard graph created by recording optical densities from serial dilutions of EB in 0.9% sodium chloride solution. The EB concentration was expressed as microgram of EB per gram of brain tissue.

The microvessel fractions were isolated from the tumor tissue by centrifugation in 15 ml with 18% (w/v) dextran solution at 10,000 *g* and 4°C for 10 min. Total RNA was extracted from the microvessel fractions with Trizol reagent (Life Technologies Corporation) according to the manufacturer’s instruction. The following experimental procedures for qRT-PCR were the same as *in vitro* experiments.

### Statistical Analysis

Statistical analysis was performed using SPSS 18.0 statistical software. Data was described as mean ± standard deviation (SD). Student’s *t*-test or One-way analysis of variance (ANOVA) followed by Bonferroni post-test was used to analyze the difference between two groups. *P* < 0.05 was considered to be statistically significant.

## Results

### MiR-330-3p Was Up-regulated in GECs and Low-Dose EMAP-II Inhibited the Expression of MiR-330-3p in GECs

**Figure 1:**
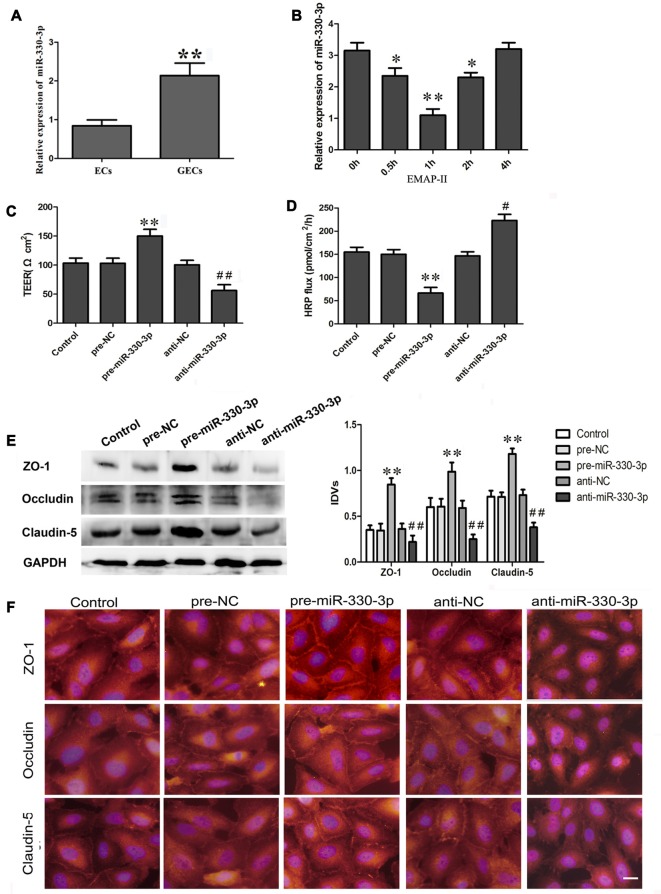
**(A)** The endogenous expression of miR-330-3p in ECs and glioma microvascular endothelial cells (GECs). U6 was used as an inner control. Data represent means ± standard deviation (SD; *n* = 5, each). **P < 0.01 vs. ECs group. **(B)** Effect of Endothelial monocyte-activating polypeptide-II (EMAP-II) on the expression of miR-330-3p in GECs. U6 was used as an inner control. Data represent means ± SD (*n* = 5, each). *P < 0.05 and **P < 0.01 vs. EMAP-II 0 h group. **(C,D)** Transendothelial electric resistance (TEER) and horseradish peroxidase (HRP) assays were used to measure the effects of overexpression or silencing of miR-330-3p on the permeability of blood–tumor barrier (BTB). **(E,F)** The expression and distribution of tight junction (TJ) related proteins in GECs after overexpression or silencing of miR-330-3p. GAPDH was used as an inner control. Data represent means ± SD (*n* = 5, each). **P < 0.01 vs. pre-NC group; ^#^*P* < 0.05 and ^##^*P* < 0.01 vs. anti-NC group.

As is shown in Figure 1A, miR-330-3p was slightly expressed in ECs. Compared with ECs, the expression of miR-330-3p was up-regulated in GECs.

As Figure 1B showed, the expression of miR-330-3p was down-regulated in GECs after EMAP-II administering 0.5 h, 1 h and 2 h. The lowest value appeared at 1 h. This result suggested that EMAP II inhibited the expression of miR-330-3p in GECs.

### Silencing of MiR-330-3p Impaired the Integrity, Increased the Permeability of BTB, and Decreased the Expression of TJ Related Proteins in GECs

After being treated with miR-330-3p, TEER assay revealed that the TEER value of GECs in pre-miR-330-3p group was increased compared with the pre-NC group, while decreased in anti-miR-330-3p group (Figure 1C). HRP flux test results were shown in Figure 1D, overexpression of miR-330-3p decreased the HRP flux of GECs, and silencing of miR-330-3p increased the HRP flux of GECs. The above results indicated that silencing of miR-330-3p could impair the BTB integrity and increase the permeability of BTB.

To clarify the potential mechanisms of miR-330-3p regulating the permeability of BTB, the protein expression levels of ZO-1, occludin and claudin-5 in GECs were measured by Western blot assay (Figure 1E). Results demonstrated that the protein expression levels of ZO-1, occludin and claudin-5 showed no significant differences among the control, pre-NC and anti-NC groups. The protein expression levels of these proteins were significantly up-regulated in the pre-miR-330-3p group compared with pre-NC group, whereas those were significantly down-regulated in the anti-miR-330-3p group compared with anti-NC group.

Immunofluorescence analysis (Figure 1F) revealed that ZO-1, occludin and claudin-5 exhibited a continuous distribution along cell border of the GECs in the control, pre-NC and anti-NC groups. In pre-miR-330-3p group, ZO-1, occludin and claudin-5 were mainly distributed on the cell-cell boundaries and were abundant in expression. However, discontinuous distribution of them was observed in anti-miR-330-3p group. Similarly, results of immunofluorescence also confirmed that the expression levels of occludin, ZO-1 and claudin-5 were significantly decreased in the anti-miR-330-3p group compared with anti-NC group.

### EMAP-II Increased the Permeability of BTB by Down-regulating MiR-330-3p

**Figure 2:**
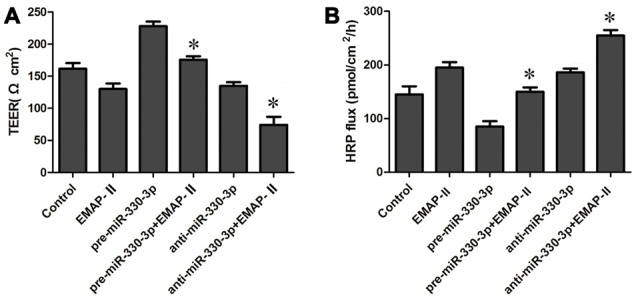
Effects of overexpression or silencing of miR-330-3p on the permeability of BTB in EMAP-II treated GECs. **(A,B)** TEER and HRP assays were used to measure the permeability of BTB. Data represent means ± SD (*n* = 5, each). *P < 0.05 vs. EMAP-II group.

In order to test the effect of miR-330-3p on EMAP-II increasing the permeability of BTB, GECs were treated with EMAP-II after miR-330-3p overexpressing or silencing. As is shown in Figure 2A, the TEER value was significantly increased in pre-miR-330-3p+EMAP-II group compared with the EMAP-II group. This meant miR-330-3p overexpressing could block the effect of EMAP-II decreasing the TEER value. On the contrary, the TEER value was significantly decreased in anti-miR-330-3p+EMAP-II group compared with the EMAP-II group. This implied miR-330-3p silencing could enhance the effect of EMAP-II decreasing the TEER value. The results of HRP flux assay were similar to that of TEER experiments. The HRP flux was decreased in the pre-miR-330-3p+EMAP-II group, while increased in the anti-miR-330-3p+EMAP-II group (Figure 2B). These results revealed that EMAP-II increased the permeability of BTB by down-regulating miR-330-3p.

### PKCα Was a Target Gene of MiR-330-3p

**Figure 3:**
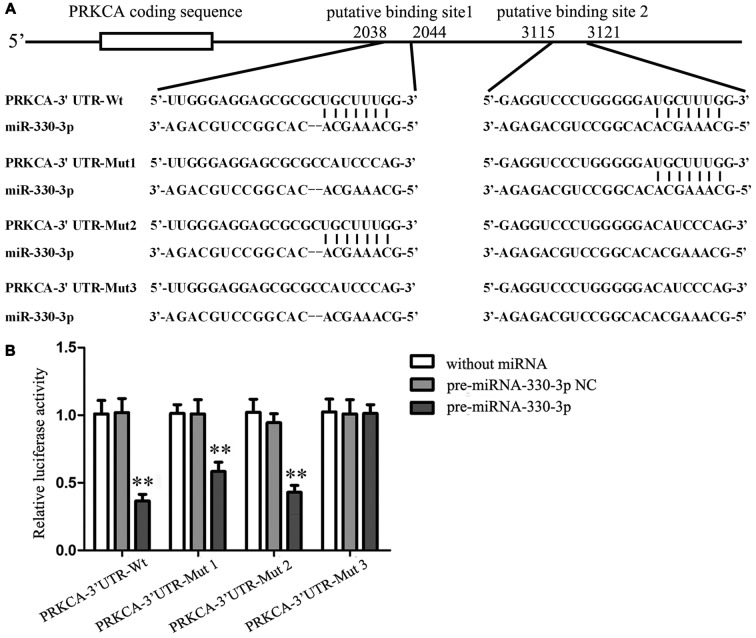
PKCα was a target gene of miR-330-3p. **(A)** The putative binding sites of PKCα 3′UTR (PRKCA-3′UTR) matching with the seed region of miR-330-3p were predicted with the help of TargetScan and the site mutagenesis design for the reporter assay (PRKCA-3′UTR-Mut1, PRKCA-3′UTR-Mut2, and PRKCA-3′UTR-Mut3).** (B)** Relative luciferase activity was expressed as firefly/renilla luciferase activity. Values are means ± SD (*n* = 5, each). **P < 0.01 vs. pre-miR-330-3p-NC.

To elucidate the molecular mechanisms of miR-330-3p regulating PKCα expression, the potential targets of miR-330-3p was predicted by using the TargetScan Human Release 6.2. The results showed that the 3′ UTR of PKCα mRNA contains two putative binding sites, which shared the same seed region with miR-330-3p (Figure 3A). To verify that PKCα was a functional target of miR-330-3p, we cloned reporter plasmids containing the wild-type 3′UTR of PKCα (PRKCA-3′UTR-Wt) and three mutant-type 3′UTR of PKCα (PRKCA-3′UTR-Mut1/Mut2/Mut3). The mutant-type reporter plasmids were constructed to determine the regions responsible for miR-330-3p, where two seed sequences of miR-330-3p were mutated individually (PRKCA-3′UTR-Mut1 and PRKCA-3′UTR-Mut2) or were mutated in combination (PRKCA-3′UTR-Mut3). As is shown in Figure 3B, cotransfection of pre-miR-330-3p and PRKCA-3′UTR-Wt significantly decreased the luciferase activity, while cotransfection of pre-miR-330-3p NC and PRKCA-3′UTR-Wt did not change the luciferase activity. The luciferase activities were markedly diminished in cells transfected with pre-miR-330-3p and PRKCA-3′UTR-Mut1/Mut2, compared with cells transfected with pre-miR-330-3p-NC and PRKCA-3′UTR-Mut1/Mut2 respectively. However, there was no significant difference in PRKCA-3′UTR-Mut3 group. These results showed that miR-330-3p functioned as a regulator of PKCα by binding to the two sites (putative binding sites 1 and 2) in PKCα 3′UTR.

### EMAP-II Up-regulated the Expression Levels of PKCα and p-PKCα via Down-regulating MiR-330-3p

**Figure 4:**
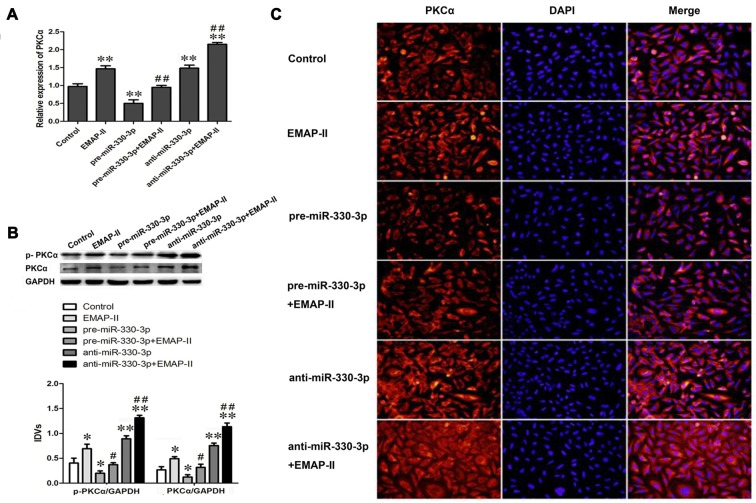
Effects of overexpression or silencing of miR-330-3p on the expression levels of PKCα and p-PKCα in EMAP-II treated GECs.** (A)** The relative mRNA expression level of PKCα was detected by real-time quantitative PCR. U6 was used as an inner control. **(B)** Western blot analysis of the PKCα and p-PKCα levels in GECs. GAPDH was used as an inner control. *P < 0.05, **P < 0.01 vs. control group; ^#^*P* < 0.05, ^##^*P* < 0.01 vs. EMAP-II group.** (C)** The expression and distribution of PKCα in GECs was analyzed by immunofluorescence assay. Scale bar represents 20 μm. Values are means ± SD (*n* = 5, each).

Our study team has demonstrated that EMAP-II can up-regulate the protein expression of PKCα, but the mechanism is unclear. In order to detect whether EMAP-II regulates PKCα by affecting miR-330-3p, the mRNA and protein expression levels of PKCα and the expression level of p-PKCα were detected after EMAP-II administering based on miR-330-3p overexpressing or silencing in GECs. As is shown in Figure 4A, the mRNA expression level of PKCα was up-regulated in EMAP-II, anti-miR-330-3p and anti-miR-330-3p+EMAP-II groups compared with control group, whereas, it was down-regulated in pre-miR-330-3p group. This result revealed that silencing of miR-330-3p could up-regulate the mRNA expression of PKCα. Compared with EMAP-II group, the mRNA expression level of PKCα was significantly down-regulated in pre-miR-330-3p+EMAP-II group and up-regulated in anti-miR-330-3p+EMAP-II.

The results of Western blot assay and immunofluorescence analysis were similar to that of qRT-PCR assay. As Figure 4B showed, EMAP-II, anti-miR-330-3p and anti-miR-330-3p+EMAP-II up-regulated the expression levels of PKCα and p-PKCα, while pre-miR-330-3p down-regulated the expression levels of them. Compared with EMAP-II group, the expression levels of PKCα and p-PKCα were significantly down-regulated in pre-miR-330-3p+EMAP-II group and up-regulated in anti-miR-330-3p+EMAP-II. As is shown in Figure 4C, PKCα was mainly expressed in the cytoplasm of GECs and the change trend of PKCα in each group was consistent with the results of RT-PCR assay and Western blot assay. These results indicated that EMAP-II up-regulated the expression and activity of PKCα via down-regulating miR-330-3p.

### MiR-330-3p Combined with PKCα Activator or PKCα Inhibitor Affects BTB Permeability and the Expression Levels of TJ Related Proteins

**Figure 5:**
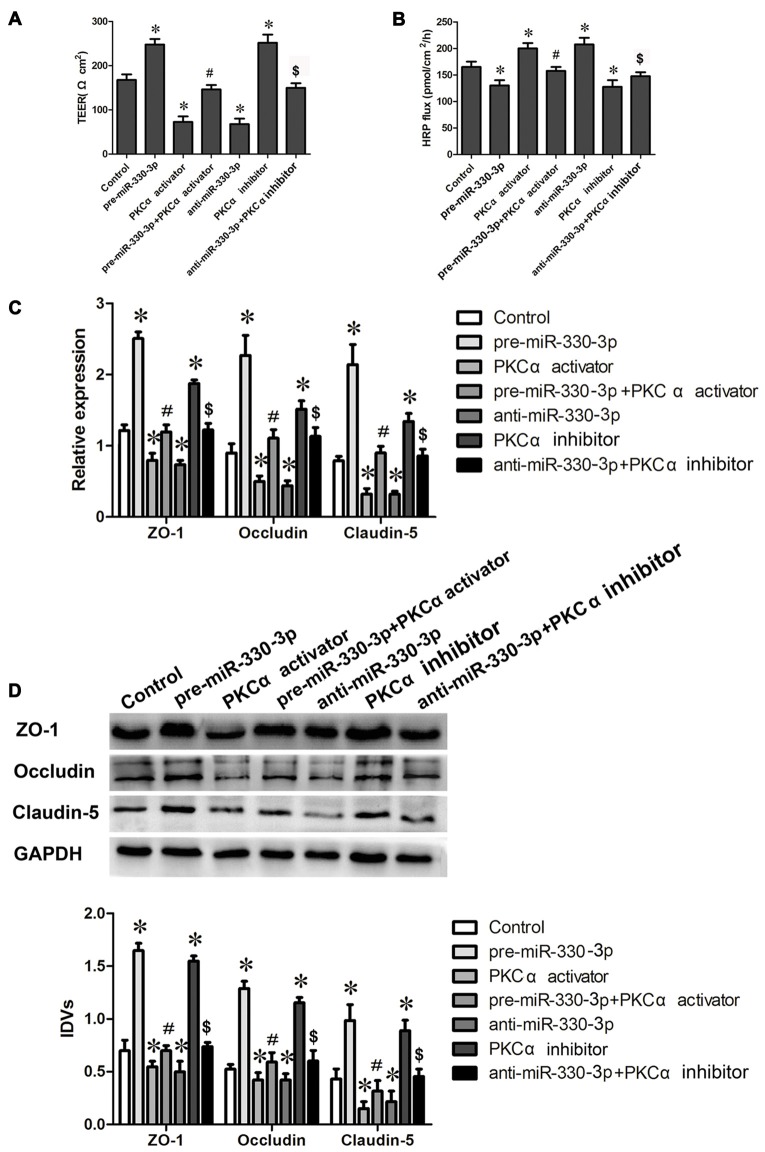
MiR-330-3p combined with PKCα activator or PKCα inhibitor affect BTB permeability and the expression levels of TJ related proteins.** (A,B)** TEER and HRP assays were used to measure the permeability of BTB. **(C)** The relative mRNA expression levels of ZO-1, occludin, and claudin-5 were detected by real-time quantitative PCR. U6 was used as an inner control.** (D)** The protein expression levels of ZO-1, occludin and claudin-5 were determined by Western blot. GAPDH was used as an inner control. Values are means ± SD (*n* = 5, each). *P < 0.05 vs. control group; ^#^*P* < 0.05 vs. pre-miR-330-3p group; ^$^*P* < 0.05 vs. anti-miR-330-3p group.

To determine the effect of PKCα activity on miR-330-3p regulating the BTB permeability and the expression levels of ZO-1, occludin and claudin-5, PKCα activator PMA and inhibitor staurosporine were used in miR-330-3p overexpression or silencing GECs. TEER and HRP flux assay were respectively investigated. As Figures 5A,B showed, the TEER values increased and HRP flux decreased in pre-miR-330-3p and PKCα inhibitor groups compared with control group. However, the TEER value decreased and HRP flux increased in PKCα activator and anti-miR-330-3p groups compared with control group. Simultaneously, pre-miR-330-3p+PKCα activator significantly decreased the TEER value and increased the HRP flux compared with pre-miR-330-3p. While, anti-miR-330-3p+PKCα inhibitor obviously increased the TEER value and decreased the HRP flux compared with anti-miR-330-3p.

In addition, the mRNA and protein expression levels of TJ related proteins ZO-1, occludin and claudin-5 were further detected by qRT-PCR and Western blot assays. The results showed that the change trend of TJ related proteins expression was consistent with the trend of TEER values (Figures 5C,D). Compared with control group, the mRNA and protein expression levels of ZO-1, occludin and claudin-5 increased in pre-miR-330-3p and PKCα inhibitor groups, while decreased in PKCα activator and anti-miR-330-3p groups. Compared with pre-miR-330-3p, pre-miR-330-3p+PKCα activator significantly decreased the mRNA and protein expression levels of ZO-1, occludin and claudin-5. Compared with anti-miR-330-3p, anti-miR-330-3p+PKCα inhibitor obviously increased the mRNA and protein expression levels of them. These results revealed that miR-330-3p could affect the BTB permeability and the expression levels of TJ related proteins by regulating PKCα activity.

### EMAP-II Combined with Anti-miR-330-3p and PKCα Activator Could Increase BTB Permeability and the Expression Levels of TJ Related Proteins in GECs

**Figure 6:**
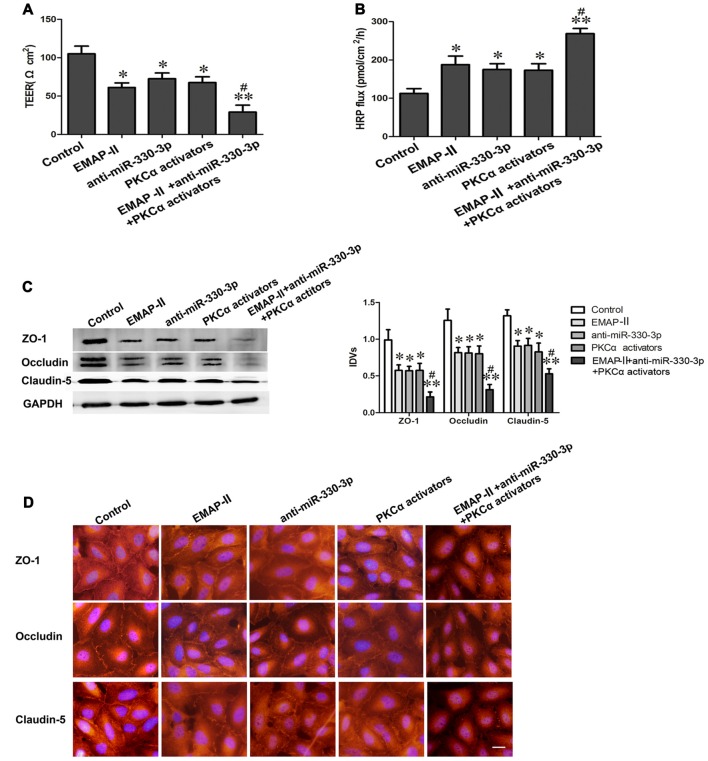
EMAP-II combined with anti-miR-330-3p and PKCα activator increases BTB permeability and the expression levels of TJ related proteins in GECs.** (A,B)** TEER and HRP assays were used to measure the permeability of BTB. **(C)** The protein expression levels of ZO-1, occludin and claudin-5 were determined by Western blot. GAPDH was used as an inner control. Values are means ± SD (*n* = 5, each). *P < 0.05, **P < 0.01 vs. control group; ^#^*P* < 0.05 vs. EMAP-II group.** (D)** The expression and distribution of ZO-1, occludin and claudin-5 in GECs was analyzed by immunofluorescence assay. Scale bar represents 20 μm.

As is shown in Figures 6A,B, the TEER values decreased and HRP flux increased in EMAP-II, anti-miR-330-3p, PKCα activator and EMAP-II+anti-miR-330-3p+PKCα activator groups compared with control group. EMAP-II+anti-miR-330-3p+PKCα activator obviously decreased the TEER values and increased the HRP flux compared with EMAP-II, anti-miR-330-3p and PKCα activator, respectively.

In addition, the expression and distribution of ZO-1, occludin and claudin-5 were further detected by Western blot assay (Figure 6C) and immunofluorescence analysis (Figure 6D). The results showed that ZO-1, occludin and claudin-5 had certain expression and exhibited a continuous distribution along cell border in GECs in the control group. Compared with the control group, the protein expression levels of TJ related proteins were down-regulated and distributed discontinuously in EMAP-II, anti-miR-330-3p, PKCα activator and EMAP-II+anti-miR-330-3p+PKCα activator group, and the change of EMAP-II+anti-miR-330-3p+PKCα activator group was the most obvious.

All above results suggested that EMAP-II combined with anti-miR-330-3p and PKCα activator could increase the permeability of BTB effectively.

### EMAP-II Combined with Anti-miR-330-3p and PKCα Activator Could Enhance the Effects of DOX on Inhibiting the Cell Viabilities and Promoting the Apoptosis of U87 Glioma Cells

**Figure 7:**
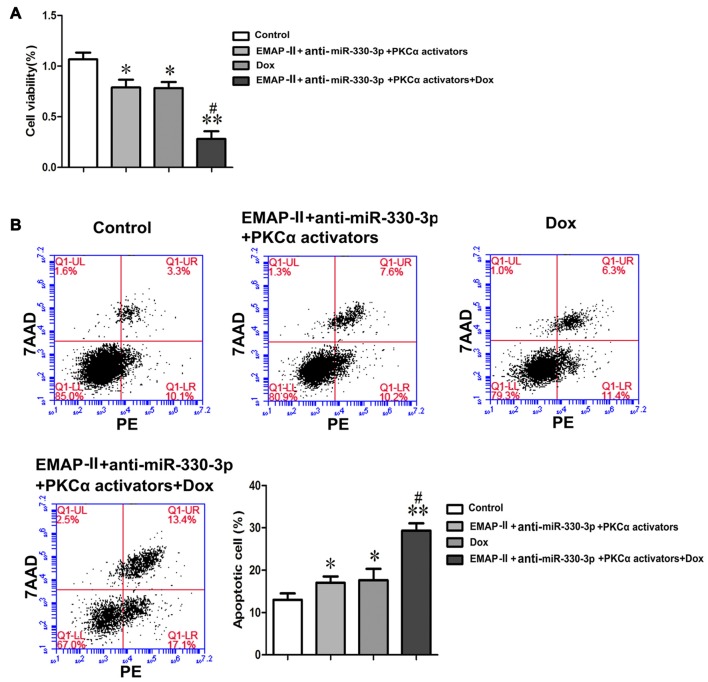
EMAP-II combined with anti-miR-330-3p and PKCα activator enhance the effects of DOX on inhibiting the cell viabilities and promoting the apoptosis of U87 glioma cells.** (A)** The cell viabilities of U87 glioma cells were assessed by MTT. **(B)** Apoptosis analysis inU87 glioma cells was evaluated by Annexin V-PE/7-AAD staining. Values are means ± SD (*n* = 5, each). *P < 0.05, **P < 0.01 vs. control group; ^#^*P* < 0.05 vs. DOX group.

As Figure 7A showed, compared with the control group, the cell viabilities of U87 cells were inhibited in EMAP-II+anti-miR-330-3p+PKCα activator, DOX and EMAP-II+anti-miR-330-3p+PKCα activator+DOX groups. The cell viability of EMAP II+anti-miR-330-3p+PKCα activator+DOX group was significantly decreased compared with DOX group. These results suggested EMAP-II combined with anti-miR-330-3p and PKCα activator could enhance the effect of DOX on inhibiting the cell viabilities of U87 glioma cells.

Meanwhile, we detected the apoptosis rate of these groups in U87 glioma cells. Similar to the result of cell viability, the apoptosis rate of EMAP-II +anti-miR-330-3p+PKCα activator, DOX and EMAP II+anti-miR-330-3p+PKCα activator+DOX groups were increased compared with the control group. The apoptosis rate of EMAP-II+anti-miR-330-3p+PKCα activator+DOX group was significantly increased compared with the DOX group (Figure 7B).

### EMAP-II Increased the BTB Permeability *in Vivo* via Down-regulating MiR-330-3p Orthotopic Xenograft Model

**Figure 8:**
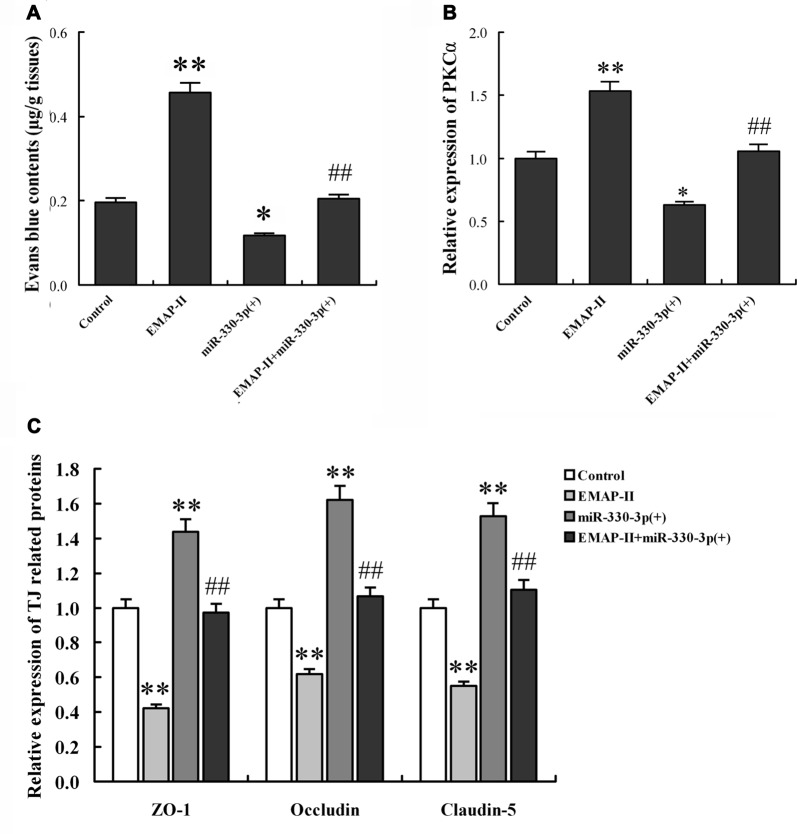
EMAP-II increased the BTB permeability *in vivo* via down-regulating miR-330-3p orthotopic xenograft model.** (A)** Contents of evans blue (EB) in tumor-bearing brain after EMAP-II and miR-330-3p lentivirus injection. The expression levels of PKCα **(B)** and TJ proteins **(C)** in tumor microvessel segments were detected by qRT-PCR. Values are means ± SD (*n* = 5, each). *P < 0.05, **P < 0.01 vs. control group; ^##^*P* < 0.01 vs. EMAP-II group.

To determine the functional role of miR-330-3p on EMAP-II increasing the BTB permeability *in vivo*, The BALB/c athymic nude mice were received an intracerebral injection of U87 glioma cells into the right striatum. Effect on BTB permeability for EB extravasation showed that the brain tumor tissue of orthotopic xenograft model was stained in blue in EMAP-II group, while no visible staining was found in control, miR-330-3p (+) and EMAP-II+miR-330-3p (+) groups. Compared with control group, the EB content of tumor-bearing brain significantly increased after EMAP-II administering and decreased after miR-330-3p (+) lentivirus injection, while there was no change in EMAP-II+miR-330-3p (+) group. Compared with EMAP-II group, the EB content was significantly decreased in EMAP-II+miR-330-3p (+) group. This result revealed that miR-330-3p overexpression blocked the effect of EMAP-II on increasing BTB permeability (Figure 8A).

The expression of PKCα, ZO-1, occludin and claudin-5 in tumor microvessel segments was detected by Real-time PCR assay (Figures 8B,C). The results demonstrated that the expression of PKCα was significantly up-regulated in the EMAP-II group and down-regulated in miR-144 (+) group compared with the control group, however, there was no change in EMAP-II+miR-330-3p (+) group. Compared with EMAP-II group, the expression of PKCα was significantly decreased in EMAP-II+miR-330-3p (+) group. MiR-330-3p overexpression blocked the effect of EMAP-II on increasing the expression of PKCα. On the contrary, the expression of ZO-1, occludin and claudin-5 were decreased in EMAP-II group and increased miR-330-3p (+) group. MiR-330-3p overexpression blocked the effect of EMAP-II on decreasing the expression of ZO-1, occludin and claudin-5.

In a word, EMAP-II increased the BTB permeability and PKCα expression, as well as decreased ZO-1, occludin and claudin-5 expression via down-regulating miR-330-3p.

## Discussion

In the present study, we found that miR-330-3p was over-expressed in GECs and EMAP-II could down-regulate the expression of miR-330-3p. Silencing of miR-330-3p increased the permeability of BTB and decreased the protein expression levels of ZO-1, occludin and claudin-5. EMAP-II could increase the permeability of BTB via down-regulating miR-330-3p in GECs. MiR-330-3p bound to the 3′UTR of PKC-α to inhibit the expression and activity of PKC-α. *In vitro* and *in vivo* results showed that EMAP-II could down-regulate the expression of miR-330-3p to up-regulation the expression and activity of PKC-α, which could reduce the expression of TJ related proteins and increase the permeability of BTB. EMAP-II combined with anti-miR-330-3p and PKC-α activator significantly increased the permeability of BTB and enhanced the effects of DOX on inhibiting the cell viabilities and promoting the apoptosis of U87 glioma cells.

There are two pathways of drugs being delivered into the tumor cells through the BTB including paracellular pathway and transcellular pathway (Xie et al., 2012) Paracellular pathway refers to the transport of substances from the blood vessels to the brain through opening TJ between microvascular endothelial cells (Abbott et al., 2010; Haseloff et al., 2015). TJ complex is composed of transmembrane proteins (occludin and claudins) and cytoplasmic proteins (zonula occludens, ZOs), which anchor to actin-based cytoskeleton proteins to form a closed structure (Wolburg et al., 2009). When the expressions of TJ related proteins ZO-1, occludin and claudin-5 were decreased, the TJ was opened and the permeability of BTB was increased (Drapeau and Fortin, 2015; Wang et al., 2015).

EMAP-II is a multifunctional polypeptide with proinflammatory and antiangiogenic activity. EMAP-II regulates the function of endothelial cells and monocytes, induces apoptosis in tumors and inhibits tumor angiogenesis (Mogylnytska, 2015). Our researches has confirmed that low-dose EMAP II increases the permeability of BTB selectively by down-regulating TJ related proteins in brain microvascular endothelial cells without affecting normal brain in a time-dependent manner (Xie et al., 2010). Studies have demonstrated that miRNAs, one of the non coding RNAs, play an important role in regulating the permeability of BTB. For example, miR-18a increased the permeability of BTB via MEF2D and RUNX1 mediated down-regulation of ZO-1, occludin and claudin-5 (Miao et al., 2015; Zhao Y.-Y. et al., 2015). MiR-144 regulated the permeability of BTB through negatively regulating the expression of HSF2 to affect the expression of ZO-1, occludin and claudin-5 (Cai et al., 2015). However, whether EMAP-II opened the TJ to increase the permeability of BTB by regulating the expression of miRNAs is unclear.

Studies have indicated that miR-330 performs different functions in various tumor tissues and cells. MiR-330 might be oncogenes in some tumors while act as a tumor suppressor gene in other tumors. MiR-330-3p, as one of the important subtypes of miR-330, plays different roles in different tissues. Studies have shown that miR-330-3p plays an oncogenes role in breast cancer, ESCC, NSCLC and glioma (Qu et al., 2012; Yao et al., 2014; Liu X. et al., 2015; Meng et al., 2015; Mesci et al., 2017). However, the roles of miR-330-3p in the regulation of vascular endothelial cells and TJ in BTB, and in EMAP-II enhancing BTB permeability have not been reported. This study found that miR-330-3p was over-expressed in GECs and EMAP-II could down-regulate the expression of miR-330-3p in a time-dependent manner. The lowest point of miR-330-3p expression appeared at 1 h after EMAP-II administering, which was consistent with the time that EMAP-II down-regulated the expression levels of TJ related to the minimum value and increased the permeability of BTB to the peak (Xie et al., 2010). This indicated that miR-330-3p might be involved in the process in which EMAP-II regulated the permeability of BTB by opening the TJ. In this respect, we tested the effect of miR-330-3p on regulating the permeability of BTB and the expression of TJ related proteins in GECs. The results showed that silencing of miR-330-3p down-regulated the protein expression levels of TJ related proteins ZO-1, occludin and claudin-5 and increased the permeability of BTB. These results suggested that silencing of miR-330-3p increased the permeability of BTB by paracellular pathway. Meanwhile, we found that EMAP-II’s decreasing the TEER values or increasing the HRP flux was enhanced by miR-330-3p silencing, while blocked by miR-330-3p overexpression. Combining the above findings, we concluded that EMAP-II increased the permeability of BTB by down-regulating miR-330-3p.

MiRNAs can directly degrade mRNA or inhibit its post transcriptional level in mammalian by targeting binding the 3′UTRs region of mRNAs, which is involved in the regulation of gene expression (de Moor et al., 2005; Robins and Press, 2005). Protein kinase C (PKC) is an important molecule that regulates the dynamic changes of TJ and affects the permeability of endothelial cells. It is well known that the PKC is categorized into three distinct subgroups: the Ca^2+^-dependent conventional PKCs (α, β and γ), the Ca^2+^-independent novel PKCs (ε, δ, θ, η and μ), and the Ca^2+^-independent atypical PKCs (λ and ζ). Study has reported that three PKC isoforms, PKC-α, β and ζ, are involved in the process in which EMAP-II increased the BTB permeability through regulating TJ function of brain microvascular endothelial cells, without involvement of other types (Li et al., 2012a, 2015b).

To clarify the role of miR-330-3p in EMAP-II regulating PKC-α, the expression and activity of PKC-α were detected after EMAP-II administering based on miR-330-3p overexpressing or silencing in GECs. The results showed that silencing of miR-330-3p enhanced the EMAP-II’s effect on increasing the expression levels of PKC-α and p-PKC-α, while overpression of miR-330-3p blocked the above effect of EMAP-II. Bioinformatics TargetScan Human Release 6.2 analysis implied that PKC-α might be a target gene of miR-330-3p. Dual-luciferase reporter assay verified that miR-330-3p could target bind to 3′UTR of PKC-α mRNA. According to the above results, we considered that EMAP-II enhanced the expression and activity of PKC-α via down-regulating miR-330-3p.

In order to verify whether miR-330-3p regulated BTB permeability by altering PKCα activity, PKCα specific activator or inhibitor were used in miR-330-3p overexpression or silencing GECs. The findings implied that PKCα is activated after phosphorylation, and the activated PKCα up-regulates the expression of TJ related proteins, leading to the opening of TJ, ultimately increasing the permeability of BTB. PKC is a serine/threonine protein kinase, and different PKC isoforms are involved in different pathological processes by regulating TJ related proteins. Studies have found that down-regulation of claudin-1 expression is induced by TNF-α is regulated by the PKCδ–iPLA2–PGE2–PPARγ signaling cascade in human lung carcinoma A549 cells, which caused the change of morphology and migration ability (Iitaka et al., 2015). EMAP-II increases BTB permeability by activating PKC-α and PKC-β (Li et al., 2015c). TJ related proteins ZO-1, occludin and claudin-5 have a plurality of PKC phosphorylation sites. Some subtypes of PKC activation can alter the phosphorylation status, induce redistribution of TJ related proteins by phosphorylating them, and then play a role in directly regulating TJ, and increasing the permeability of the blood-brain barrier (Sjö et al., 2010; Willis et al., 2010; Li et al., 2016). These revealed that PKC-activation increases the permeability of BTB by phosphorylating ZO-1, occludin and claudin-5. According to the above results, we have concluded that miR-330-3p affects the permeability of BTB by regulating the expression and activity of PKC-α. Similar to our results, it has been reported that miR-34 regulates BTB permeability by target controlling the expression and activity of PKCε (Zhao W. et al., 2015).

This research finally proved that EMAP-II combined with anti-miR-330-3p and PKC-α activator significantly reduced the expression and altered the distribution of TJ related proteins ZO-1, occludin and claudin-5, and increased the permeability of BTB. In addition, we found that EMAP-II combined with anti-miR-330-3p and PKC-α activator could enhance the effects of DOX on inhibiting the cell viability and promoting apoptosis of glioma cells. DOX exhibited a high anti-tumor effect against the glioblastoma, inhibited the growth of U87 glioma cells and was used to evaluate the anti-glioma effects of various drugs across the BTB (Zheng et al., 2015; Chen et al., 2017; Malinovskaya et al., 2017). Therefore, our study revealed that the combination of EMAP-II with other anti-glioma drugs may be a new way of comprehensive treatment of glioma.

Except PKC-α, miR-330-3p has many other targets that may be relevant, such as Cystatin C and AKT3. Study showed cystatin C was involved in the process of VEGF-inducing angiogenesis and enhanced neuronal autophagy in neurovascular units (Wang et al., 2017; Zou et al., 2017). PI3K/Akt signaling is an important pathway for autophagy and has neuroprotective effects (Xu et al., 2013; Ma et al., 2015). These molecules might play important roles in EMAP-II regulating the function of GECs and glioma cells biological activity. Therefore, the role of multiple target genes downstream of miR-330-3p was explored to provide a more comprehensive analysis of EMAP-II increasing the permeability of BTB by down-regulating miR-330-3p.

**Figure 9:**
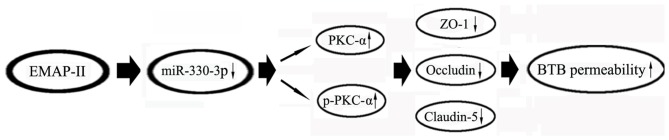
The schematic cartoon of EMAP-II increasing the BTB permeability.

In summary, our present study first demonstrates that low-dose EMAP-II-mediated opening of BTB by firstly inhibiting the expression of miR-330-3p, then up-regulating the expression and the activity of PKC-α, and finally reducing the TJ related proteins ZO-1, occludin and claudin-5. The combination of EMAP-II, anti-miR-330-3p and PKC-α activator significantly increased the BTB permeability and enhanced the antitumor effects of DOX on the glioma cells. The mechanism of EMAP-II increasing the permeability of BTB is schematically presented in Figure 9. The results of this study provide a new experimental basis for EMAP-II combined with other anti-glioma drugs to improve the chemotherapy effect of glioma.

## Author Contributions

YX, YL and LL: conceived and designed the experiments. JL and LL: performed the experiments. SC, XL, JZ, JC and WG: analyzed the data. HT and ZL: contributed reagents/materials/analysis tools. JL, LL, PW and YX: wrote the manuscript.

## Conflict of Interest Statement

The authors declare that the research was conducted in the absence of any commercial or financial relationships that could be construed as a potential conflict of interest.
